# Surgical Approaches to Benign Parapharyngeal Space Tumors-5-Year Experience

**DOI:** 10.22038/ijorl.2019.38350.2268

**Published:** 2020-01

**Authors:** Keyvan Aghazadeh, Khorsandi Mohammadtaghi, Mohamamadjavad Rikhtegar, Amirsina Sharifi, Arsalan Hashemiaghdam, Arezou Hashem Zade

**Affiliations:** 1 *Otorhinolaryngology Research Center, * *Tehran University of Medical Sciences, Tehran, Iran.*; 2 *Students’ Scientific Research Center, Faculty of Medicine, Tehran University of Medical Sciences, Tehran, Iran.*

**Keywords:** Para-pharyngeal space tumor, Pleomorphic adenoma, Transoral approach, Tanscervical approach

## Abstract

**Introduction:**

Various surgical approaches to parapharyngeal space (PPS) tumors are introduced to obtain complete removal with the preservation of the surrounding structures in parapharyngealneoplasms. Here, we will discuss the main techniques and their outcome.

**Materials and Methods:**

This retrospective study was conducted on 78 patients undergone either transoral, transcervical or a combination of these two approaches for the resection of PSS tumors from January 2010 to January 2015.

**Results:**

A number of 33 male and 45 female patients with the mean age of 40.9 ± 9.1 were evaluated. 42.3% of the patients were asymptomatic at the initial presentation. Pleomorphic adenoma and schwannoma were a permanent diagnosis in 61(78.2%) and 11(14.1%) patients, respectively. PPS tumors were resected using transoral, transcervical and combined approaches in 35(44.8%), 33(42.3%) and 10 (12.9%) cases, respectively. Recurrence occurred in 10 patients all of whom had apre-styloid pleomorphic adenoma, operated transcervical (P< 0.0001).Three cases of tenth nerve palsy occurred in schwannomas which were operatedtranscervically (P=0.04). Mean hospital stays were 2.11,3.69, and 4.9 days after transoral, transcervical and combined approaches, respectively (P= 0.001).

**Conclusion:**

Transoral, transcervical and combined approaches are all able to provide adequate visualization with comparable outcomes.

## Introduction

The para-pharyngeal space (PPS) which is described as an inverted pyramid-shaped potential spaceextending from the base of the skull to the greater cornu of the hyoid bone, has been a challenging area to access safelyeven for the expert surgeons (1,2). Accordingly, the operation-related complications are higher in these cases due to the rich vascular and neural structures in this area (3). Tumors of PPS are rare, accounting for only 0.5% of all head and neck neoplasm’s and80% of tumors originated from this area are benign (4), and the most common origins are salivary and neurogenic (5). Surgery is a  mainstay of treatment for PPS tumors pursuing four significant goals, including complete tumor removal, function preservation, minimal morbidity, and satisfactory cosmetic outcomes (6). To this end, different surgical access routehasbeen developed, namelytransoral, transcervical or transparotid, trans-mandibular and a combination of these methods (5,7-9). Access routeshould be selected according to tumor characteristics, such as size, critical relationships, and natural behavior. Moreover,the surgeon’s preference and experience may influence the surgical approach (10).The currentstudyreported our five-year experience of PPS tumor resection, and compared the transoral approach, transcervical approach, and a combination of these them in the resection of various types of PPS tumors.

## Materials and Methods

This prospective analysiswas conducted on all theconsecutive patients with PPS referred to Amir Alam Hospital, Tehran University of Medical Sciences, Tehran, Iran from January 2010 to January 2015. Institutional Review Board and the Ethics Committee of Amir Alam Hospital approved the study protocol. Patients who had neoplasm of elsewhere metastatic to PPS and tumors primarily treated in other centers were excluded. Moreover, those tumors which showed to be malignant either in fine needle aspiration (FNA) pathology or manifested aggressive behaviors, such as adhesion to adjacent structures were ruled out. The data were collected in such areas as age, sex, presenting symptoms, preoperative FNA, magnetic resonance imaging (MRI), pathological characteristics of the tumor, surgical approach, hospital stay, and postoperative complications. The decisions on surgical approaches for each patient were taken during tumor board sessions. The transcervical-approachwas performedon a design described by Chang et al. (10), and transoralapproach as defined by Cassoni et al. (11). Postoperative control MRI was performed at 6 and 12 months after surgery based on our protocol. The data were analyzed in SPSS Software(version22). Comparison between qualitative variables and quantitative variables was made using the two-tailed Fisher’s exact and ANOVA test, respectively. In each analysis, P-value less than was considered statistically signiﬁcant.

**Table 1:** Clinical-pathological characteristics of PSS tumors

Characteristics		Pleomorphic adenoma	Schwannoma	Lipoma	Neurofibroma	P value
Mean age		41.2±8.6	37.91±9.6	48±13.88	31.5±2.12	0.58
M:F		21:40	8:3	2:2	2:0	0.63
Common signs	Oropharyngeal swelling	31(50.8%)	4(36.4%)	0(0%)	2(100%)	0.85
	Neck swelling	24(39.3%)	7(63.6%)	4(100%)	0(0%)	0.25
Common Symptom	No symptom	23(37.7%)	6(54.5%)	4(100%)	0(0%)	0.38
	Hot potato voice	19(31.1%)	2(18.2%)	0(0%)	2(100%)	0.51
Mean largest dimension		5.74±0.92	7±0.89	6.75±0.95	5.5±0.7	0.34
Mean follow-up time		30.89± 20.02	37.09±13.63	30± 15.49	28±12.4	0.72

## Results

A number of 61 patients (78.2%) were diagnosed with pleomorphic adenoma, and preoperative FNA could diagnose 57 of them. However, the reports on four patinets turned out to beinconclusive. Schwannomawas reportedas the final pathology in 11cases(14.1%) in which preoperative FNA reported 7 cases as spindle cell, 2 cases as nuclear pleomorphism and two as inconclusive. The final pathologies off our patients (5.1%) were concluded as lipoma, and preoperative FNA results were comparable in all cases. Final pathology was suggestive of neurofibromatosis in two patients (2.6%) in which preoperative FNA reported in conclusive result. We were not able to assess the sensitivity and specificity of preoperative FNA since we ruled out those patients with suspicion of malignancy based on preoperative FNA conclusion. The comprehensive description ofsurgical approachesin each tumor is presented in (Table.2). 

**Table 2:** Surgical approaches in PSS tumors

	**Pleomorphic adenoma**	**Schwannoma**	**Lipoma**	**neurofibroma**	**Total**
Transoral	33(54.1%)	0	0	2(100%)	35(44.8%)
Transcervical	22(36.1%)	7(63.6%)	4(100%)	0	33(42.3%)
Combined	6(9.8%)	4(36.4%)	0	0	10(12.9%)

In this respect, there was no in traoperative complication and no need to expand the surgical field. Most tumors (65 cases, 83.3%) were located in the pre-styloidarea, and 13patients (16.7%) were diagnosed with tumors in the post-styloidregion. The mean size of the largest dimension of all tumors according to permanent pathology reports was 5.96±1.02, 4.23±0.97 and 2.60 ± 0.94 for length, width, and height, respectively. The mean follow-up duration was 30.9±18.8with a rangeof28-60 months.

Postoperativerecurrenceoccurred in 10 patients and all of whom had pre-styloid pleomorphic adenoma which was operatedtranscervically (P-< 0.0001). Mean of the largest dimension of pleomorphic adenoma was reported as 5.85±0.93, 5.55±1.1 and 6.33±0.51, respectivelyas if operated transorally, transcervical and combined (P= 0.19). The mean of the most extensive dimension of schwannoma was found to be 6.57±0.78 and 7.25±0.95 as if operated transcervically and in combination (P= 0.23).

Three patients were diagnosed with tenth nerve palsies who had the permanent pathology of schwannoma and were operatedtranscervically (P=0.04). Horner’s syndrome was detected in eight patients (10.3%) who had schwannomas and were operated transcervically or with combined approaches (P=0.49). There was no case with nerve 7, 9, 11 or 12 palsies. Trismus was not observed in postoperative visits. Detailed postoperative complications are presented in (Table.3).

**Table 3:** Post-operative complications in each surgical approach

	Transoral	Transcervical	Combined	P value
Nerve 7 palsy	0	0	0	-
Nerve 9 palsy	0	0	0	-
Nerve 10 palsy	0	3	0	0.04
Nerve 11 palsy	0	0	0	-
Nerve 12 palsy	0	0	0	-
Trismus	0	0	0	-
Horner’s syndrome	0	4	4	0.49
Hematoma	1	2	1	0.63
Hospital stay	2.11	3.69	4.9	0.001
PO time	2	1	2	0.1
Recurrence	0	10	0	<0.0001

Median hospital stay was 2, 3 and 5 days after transoral, transcervical and combined approaches, respectively, which indicated no significant difference (P=0.001). In addition, the subgroups were not different in terms of post-operative time to initiate postoperative regimen (P=0.1). 

## Discussion

Tumors originating from PPS are mostly benign with thesalivarysource. However, some authors argued that neurogenic sourceis the most prevalent one (12). Regardless of the PPS tumor origin, they are rarely symptomatic before reaching a diameter of at least3cm; therefore, patients are unaware of tumor presence in many cases and PPS tumor is usually found in an incidental head and neck imaging for anunrelatedreason (11). However, patients may complain about a mass in the oropharynx or upper neck, pain, trismus, change in voice, symptoms related to Eustachian tubes obstruction, odynophagia, and dysphagia (13). If PPS tumors get large enough, they might be detected as smooth submucosal mass displacing the lateral pharyngeal wall, tonsil, and soft palate in anterior aspect (13). Benign tumors of salivary origin were the most common in our patients. Regardless of tumor pathology, most of the patients did not have any symptoms related to the inaugural presentation of their tumor. 

Accurate localization of the tumor, histology prediction and relationship of mass with adjacent vital structures are achievable through MRI and/or computed tomography (CT) scan (14). An angiographic evaluation is mandatory in patients with a high suspicion of vascular tumor or dangerous proximity of the mass to the carotid vessel or even vessel invasion (15). 

As discussed before, PPS tumors are difficult to access resulting in alimited preoperative sampling of tumor cytology. Currently, transoral or transcervical FNA provides anaccurate diagnosis in 90-95% of patients, and it can be performed under CT scan or ultrasound guidance (16). However, this biopsy technique is highly operator-dependent and might be inconclusive in 25-60% of patinets (17). Core needle biopsy (CNB) provided an opportunity for the collection of a core sample of the tissue which can be used to assess tumor histopathology and immunohistochemical markers(18). The CNB is contraindicated when tumor spillage, capsule rupture, and tumor recurrence is suspected. On the other hand, FNA is not completely safe, and it might lead to excessive bleeding in vascular tumors (14). Although we excluded malignant tumors from our study, FNAwas diagnostic in 61/78of cases (78.2%). Surgical resection is the mainstay treatment in PPS tumors, and different surgical approaches havebeen defined in this respect. The choice of surgical approach relies on the tumoraccessibility, tumor pathology, and surgeon's preference (19).

The particularstrategy is implemented for a specific tumor mostly based on the presence of the tumor in pre-styloid or post-styloid spaces and tumor location on superior-inferior axis (20). Transoral approach for PSS tumor has undergone a drastic changesince 1974 after being named a blind surgical procedure by Work and Hybels et al. (21).Tumor rupture and saliva contamination of the surgical field which results in infection and delayed wound healing was claimed to be higher in transoral approach (22). However, this technique began to drew attention to anatomical barriers as Dallan et al. defined and expressed transoral approach as a first surgical window for PPS tumors (23). It turned into a promising access route in indicated patientsdue to such factors as recent technological advances in the visualization of the surgical field including Weerda laryngoscope (24), anendoscope-assisted technique by 0 and 30 telescopes and transoral robotic surgery (24,25), and low rate of postoperative complication (26). On the other hand, the main advantage of the transcervical approach is adequate exposure which provides en bloc resection in the majority of PPS tumors (27). Many researchers in different settings have recommended transcervicalapproach as the best access route despite the anatomical complexity of the PPS region (28,29). Although both benign and malignant tumors have been resected with the transcervicalapproach, the proper exposure of medial and superior aspects of PPS and hemorrhage control in vascular lesions might be challenging in trscervical approach (30). Therefore, patient allocation should be prioritized based on the potentialrisks and benefits (10). 

In the current study, 10 patients underwent combined transoral-transcervical resection of PPS tumors. These patients were designated to be operatedin this way, and surgical field expansion was not due to the lack of visualization or in traoperative complications. Betka et al. (9) used this approach in the treatment of two patients with pleomorphic adenoma of the minor salivary gland and one with schwannoma. They arrived at radical resection with no recurrence and complication in all three cases. Cassoni et al.(11) reported the same satisfactory results using the combined approach in the treatment of two patients with relapse of pleomorphic adenoma and one patient with each of the following pathologies: chemodectomas, paraganglioma, angioma, and V3 neuroma. According to our results, although it mightseem that all four schwannomas operated using a combined approach led to Horner’s syndrome, the other four schwannomas with subsequent Horner’s syndrome were operatedtranscervically without any statistically significant difference. Based on experience, hospitalstay and cosmetic outcomes are matters of concern to patients. In this respect, the patinets who underwent transoral approach had the shortest hospital stay in our study, and they seemed content with the non-development of any scars. However, the patients undergoing transcervical and a combined approach were not unsatisfied with cosmetic outcomes since only a 4-5cm scar remained. Mean hospitalization time for patients treated transcervically was 1.05 days in the study conducted by Chang et al. and seven days in another research performed by Betka et al,. which was conducted on 26 patients undergoing different transoral (9,10), transcervical and combined approaches. Recently, the transoral endoscopy-assisted method has been associated with a short hospital stay, low blood loss, and postoperative pain levels, and preserved facial cosmetic appearance.

Moreover, ten patients were found to have are currence in 5-yearfollow-upall of whom were pleomorphic adenomas treated with a transcervical approach. The relapse rate was 12.8% of benign PSS tumor in the present study, whereas this value was reported as0-9% in previous studies (8,14,31). 

## Conclusion

Based on the results of the current study, no recurrence was detected in the patients who underwent transoral approach with pleomorphic adenoma and neurofibroma tumors during follow up. Moreover, the mean hospital stay was much lower and no postoperative scar was developed, as compared to two other approaches. Although surgical management of PPS tumor still remains a challenge to surgeons, transoral approach yielded better results, compared to transcervical and combined approaches in our study, due to postoperative outcomes. We could not associate postoperative outcomes, such as tenth nerve palsy and Horner’s syndrome to transoral approachsince in our study these complications were only related to patients with schwannoma who underwent transcervical and combined approach. Transoral approach for the treatment of benign PPS tumors can be as safe as other approaches applied for these tumors. Highly selective patients protocol with complete preoperative evaluation, including MRI localization of the tumor and FNA pathology, is necessary. Furthermore, the surgeon should be well aware of the complex anatomy of the region in all surgical procedures on PPS tumors. Accordingly, a well-trained hand and watchful eyes of a surgeon play the leading role in the achievement of satisfactory outcomes. 
